# A novel radiological index for the evaluation of cervical posterior subcutaneous fat tissue thickness and cervical spine degeneration: A retrospective study

**DOI:** 10.1097/MD.0000000000034355

**Published:** 2023-07-21

**Authors:** Jian Cao, Dong Sun, Lianzhi Guo, Rui Wang, Peng Liu

**Affiliations:** a Department of Orthopaedics, China-Japan Union Hospital of Jilin University, Changchun, Jilin, China.

**Keywords:** cervical spine, intervertebral disc degeneration, neck pain, subcutaneous fat tissue thickness

## Abstract

Obesity is an important risk factor linked to the incidence of both neck pain (NP) and intervertebral disc degeneration (IVDD). Subcutaneous fat tissue thickness (SFTT) has been proposed as a more effective biomarker than body mass index (BMI) when gauging body fat levels. This study was thus designed to explore the optimal SFTT cutoff value for differentiating between NP patients and asymptomatic individuals by using the subcutaneous fat index (SFI). Magnetic resonance imaging (MRI) records from NP patients and asymptomatic controls were compared to evaluate IVDD, the fatty infiltration of the paravertebral muscles, and Modic changes. Cervical SFTT was also assessed at multiple levels. SFTT at the C3 level was found to be significantly associated with NP, with respective optimal cutoff values of 9.64 mm and 8.21 mm for females and males. Females in this study cohort more frequently exhibited spine deterioration with an SFI > 9.64 mm as compared to males with an SFI > 8.21 mm. Cervical SFTT is strongly correlated with the degree of disc degeneration. IVDD, Modic changes, and fatty infiltration in the paravertebral muscles were all more prevalent among both males and females exhibiting SFTT at the C3 level that was above the defined cutoff value.

## 1. Introduction

Neck pain (NP) is a common cause of discomfort and a leading cause of disability throughout the globe.^[1,2]^ While not the most prevalent musculoskeletal issue in the world, NP nonetheless remains a significant driver of morbidity.^[3]^ Significant correlations were seen between the prevalence of NP and female gender, obesity, and smoking.^[4]^ Cervical intervertebral disc degeneration (IVDD) is the most common cause of spinal issues including both NP as well as numbness of the upper limbs.^[5,6]^ Obesity is a growing global health concern, with a rising prevalence, and has been identified as a contributing factor to lumbar IVDD.^[7]^ IVDD is among the most common factors causing spinal pain, and NP imposes a significant economic burden with no clearly defined treatment approach.^[8]^

In 2 prior reports, NP was found to be related to patient body mass index (BMI).^[9,10]^ While BMI is widely used as an index to gauge fatty tissue levels, actual fat storage characteristics vary substantially among individuals such that BMI alone cannot accurately be used to assess the local level of adiposity in the neck region in both males and females.

Yavuz et al^[11]^ recently identified an increase in cervical adipose tissue thickness as an IVDD-related risk factor. Specifically, subcutaneous fat tissue thickness (SFTT) was closely correlated with the extent of IVDD at the C4 to C5 level. However, their study did not establish a cutoff threshold capable of differentiating between NP patients and asymptomatic controls. They also failed to explore the relationship between this finding and the degree of fat infiltration of the paraspinal muscles or Modic changes in the cervical spine. Patterns of fat deposition vary markedly between males and females, who respectively exhibit higher levels of fat accumulation in visceral and subcutaneous compartments.^[12]^ As the subcutaneous tissue is not the preferential site of fat accumulation in males, the accumulation of subcutaneous fat is generally observed preferentially in individuals who are overweight or obese.^[13]^ This study was designed to use the subcutaneous fat index (SFI) to establish the optimal SFTT cutoff value capable of differentiating between NP patients and asymptomatic controls. A secondary goal of these analyses was to assess the relative utility of this radiological indicator in light of differing gender-specific patterns of subcutaneous fat deposition.

## 2. Materials and methods

### 2.1. Patient population

This retrospective study was performed through the retrospective review of a medical database from July 2021 to July 2022. Both female and male patients 20 to 76 years of age that had attended outpatient clinics owing to chronic NP without a clearly defined cause that was triggered by neck movement, neck posture, or cervical muscle examination for at least 3 months^[14]^ and had undergone a cervical spine magnetic resonance imaging (MRI) were enrolled. In addition, asymptomatic volunteer participants 20 to 76 years of age that had not been diagnosed with NP within the last year were gathered through the Physical Examination Center of our hospital. NP patients and asymptomatic controls were excluded from this study if they had any history of prior spinal surgery, trauma, spinal infections, scoliosis, kyphosis, spinal canal stenosis (with facet joint hypertrophy, osteophyte development, ligamentum flavum flexion in degenerative cases, sagittal canal diameter <13 mm and interpedicular distance <23 mm),^[15]^ or spondylolysis. The Institutional Review Board and the Ethics Committee of China-Japan Union Hospital approved this study, which was consistent with the 1964 Declaration of Helsinki and its later revisions or other comparable ethical standards. All participants provided written informed consent.

### 2.2. MRI scanning parameters

Cervical spine MRI scans from study participants were evaluated with the Picture Archiving and Communication System. Scanning was performed with subjects in the supine position. Sagittal and axial T2WI scans was performed using a GE Discovery MR750 plus 3.0-T MRI scanner (Siemens Healthcare Sector), with the following T2WI sequence parameters: TR2500 ms, TE142 ms, a field of view 341 mm × 220 mm, matrix 320 × 320, echo time, 100 ms; repetition time, 3222 ms, layer thickness, 4 mm, layer spacing, 0.5 mm, and echo chain length, 21.

### 2.3. Grading and SFTT measurement

The Pfirrmann grading system^[16]^ was used to evaluate cervical IVDD at the C2-C3 to C6-C7 levels on T2-weighted sagittal MRI scans of the cervical spine. Pfirrmann grades I to III and IV to V were respectively considered to indicate mild-to-moderate and severe IVDD. The Modic classification system was used to assess cervical vertebral endplates from the C2-C3 to C6-C7 levels based upon T1- and T2-weighted sagittal MRI scans of the cervical spine. Modic changes were assessed as follows:^[17,18]^ Type 0, normal cervical endplate signal; Type I, signal hypointensity on T1-weighted images and hyperintensity on T2-weighted images; Type II, signal hyperintensity on T1-weighted images and hyper- or isointensity on T2-weighted images; Type III, signal hypointensity on both T1- and T2-weighted images. The Goutallier classification method was used to assess the degree of fatty infiltration of the paraspinal muscles (superficial extensor, SEA and deep extensor, DEA) from the C2-C3 to C6-C7 levels on T2-weighted axial MRI images of the cervical spine as detailed previously.^[19,20]^ Goutallier grades 0 to 1 and 2 to 4 respectively indicated that fatty infiltration was absent-to-mild and moderate-to-severe. Data were collected by independent observers and analyzed by independent statisticians blinded to patient conditions.

**Figure 1.**
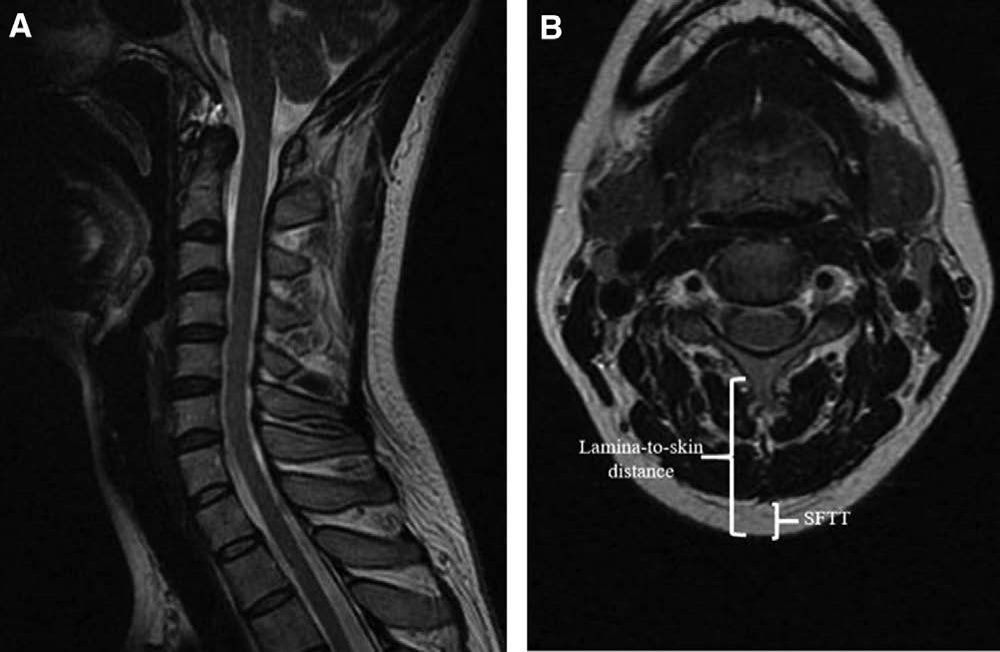
Measurement technique of SFTT at C3 level on T2-weighted axial cervical MRI. MRI = magnetic resonance imaging, SFTT = subcutaneous fat tissue thickness.

Axial T2-weighted MRI scans of the cervical spine at the C3, C5, and C7 spinous process levels were used to measure the distance of the lamina from the skin surface, paravertebral muscle thickness, and the length of the subcutaneous fat (Fig. 1). The SFI is an index used to assess the distribution and quantity of subcutaneous fat in the human body. It is typically calculated by measuring the subcutaneous fat thickness at specific body locations.

### 2.4. Statistical analysis

SPSS 22.0 (IBM, Armonk, NY) was used to conduct all statistical analyses. Categorical data were assessed using chi-square tests or Fisher exact test. Normally and non-normally distributed continuous variables were respectively analyzed with paired Student t tests and non-parametric tests. The data was not normally distributed according to the Kolmogorov–Smirnov test. The Mann–Whitney *U* test is used for non-parametric testing of 2 independent samples. *P* < .05 was the threshold of statistical significance. Area under the curve (AUC) and cutoff values were determined based on receiver operating characteristic curves with corresponding sensitivity and specificity values. Correlative relationships among variables were explored through binary logistic regression analyses, and results were reported as odds ratios (ORs) with 95% confidence intervals (CIs).

## 3. Results

### 3.1. Participant demographics and SFTT measurements

In total this study included 349 participants (155 females and 194 males; mean age: 44.3 ± 13.4 years, range: 20–76 years). The mean SFTT of these participants at the C3, C5, and C7 levels was 9.40 ± 4.25 mm, 13.66 ± 7.63 mm, and 19.37 ± 7.84 mm, respectively, while the mean lamina-to-skin distance at these respective levels was 38.63 ± 7.61 mm, 44.34 ± 7.97 mm, and 48.20 ± 7.58 mm, and the mean ratio of SFTT to lamina-to-skin distance was 0.24 ± 0.10, 0.30 ± 0.13, and 0.37 ± 0.11.

This study cohort included 200 NP patients (females: 88, males: 112; mean age: 44.4 ± 13.7 years, range: 20–76 years) and 149 asymptomatic controls (67 females, 82 males; mean age: 44.2 ± 12.9, range: 21–70). NP patients exhibited higher SFTT levels at the C3 and C5 levels, although the difference at the C7 level was not significant (*P* > .05). Differences in the lamina-to-skin distance between these 2 groups were only evident at the C5 level, with this distance being greater in NP patients (45.15 ± 8.88 mm vs 43.26 ± 6.42; *P* < .05). Consistently, NP patients exhibited a larger ratio of SFTT to lamina-to-skin distance at the C3 and C5 levels (Table 1).

**Table 1:** Demographics and measurement of subcutaneous fat tissue thickness.

	Patients	Control	*P* value
N	200	149	
Age (yr) Sex: M/F	44.4 ± 13.7 112/88	44.2 ± 12.9 82/67	.870 .857
Subcutaneous fat tissue thickness (mm) C3 C5 C7	10.10 ± 4.52 14.69 ± 8.41 19.34 ± 8.50	8.46 ± 3.65 12.28 ± 6.21 19.41 ± 6.91	<.05 <.05 .935
Lamina-to-skin distance (mm) C3 C5 C7	38.48 ± 6.55 45.15 ± 8.88 48.88 ± 7.87	38.84 ± 6.55 43.26 ± 6.42 47.30 ± 7.10	.658 <.05 .054
Ratio of fat thickness to lamina-skin distance C3 C5 C7	0.26 ± 0.11 0.31 ± 0.15 0.38 ± 0.12	0.21 ± 0.08 0.28 ± 0.13 0.40 ± 0.11	<.05 <.05 .093

Values are given as the mean or percentage and the 95% confidence interval.

### 3.2. SFI cutoff value establishment

When differentiating between the NP and asymptomatic control participants, the SFTT values at the C3 and C5 levels yielded respective AUC values of 0.595 (95%CI: 0.535–0.655, *P* < .05) and 0.566 (95%CI: 0.506–0.626, *P* < .05). The AUC value for SFTT at the C7 level was not statistically significant (0.476, 95%CI:0.415–0.537; *P* > .05). Accordingly, further analyses were only performed for SFTT values at the C3 level.

When predicting the NP status of a given individual, SFTT at the C3 level exhibited an OR of 2.119 (95% CI: 1.376–3.264, *P* < .05) at the cutoff level, with respective ORs for females and males of 2.526 (95% CI: 1.322–4.828, *P* < .05) and 2.238 (95% CI: 1.210–4.138, *P* < .05). The cutoff value of SFTT is determined according to Youden index, Youden index = sensitivity + specificity -1. The results are ranked and the maximum value of the Youden index is selected as the best critical point. Females with an SFTT of >9.64 mm and males with an SFTT of >8.21 mm exhibited significantly higher rates of severe IDD, Modic changes, and paraspinal muscle fat deposition (Tables 2 and 3). Meanwhile, we also investigated the differences in SFTT at the C3 level based on age groups. The mean SFTT at C3 for patients under the age of 65 was 9.24 ± 3.19 mm, while the mean SFTT at C3 for patients aged 65 and above was 12.49 ± 2.87 mm. Patients under the age of 65 with an SFTT value >8.49 millimeters and patients aged 65 and above with an SFTT value >12.63 millimeters exhibited more severe cervical IVDD and Modic changes (Table 4).

**Table 2:** Modic change and Intervertebral disk degeneration status of the cervical spine according to SFI cutoff value.

	Men		*P* value	Women		*P* value
	<8.21 mm	≥8.21 mm		<9.64 mm	≥9.64 mm	
For Modic change						
C2–3	0 (0.0%)	1 (0.7%)	.509	1 (1.1%)	1 (1.6%)	.786
C3–4	2 (3.4%)	17 (12.6%)	<.05	4 (6.5%)	9 (14.3%)	.108
C4–5	4 (6.8%)	23 (17.0%)	.058	8 (8.7%)	10 (15.9%)	.171
C5–6	7 (11.9%)	35 (25.9%)	<.05	11 (12.0%)	15 (24.7%)	<.05
C6–7	3 (5.1%)	15 (11.1%)	.183	9 (9.8%)	12 (19.0%)	.098
For IVDD C2–3 C3–4 C4–5 C5–6 C6–7	0 3 (5.1%) 9 (15.3%) 9 (15.3%) 8 (13.6%)	0 16 (11.9%) 38 (28.6%) 40 (29.6%) 36 (26.7%)	— .143 <.05 <.05 <.05	0 5 (4.3%) 8 (8.7%) 10 (10.9%) 3 (3.3%)	0 8 (14.3%) 10 (15.9%) 14 (22.2%) 8 (12.7%)	— <.05 .171 .055 <.05

Values in parentheses represent percentage of patients with SFI either as < cutoff or ≥ cutoff.

IVDD = intervertebral disk degeneration, SFI = subcutaneous fat index.

**Table 3:** Fatty infiltration status of the cervical paraspinal muscles according to SFI cutoff value.

		Men		*P* value	Women		*P* value
		<8.21 mm	≥8.21 mm		<9.64 mm	≥9.64 mm	
Paraspinal muscle C2–3	SEA DEA	2 (3.4%) 9 (15.3%)	4 (3.0%) 36 (26.7%)	.509 .083	5 (5.4%) 11 (12.0%)	6 (9.5%) 10 (15.9%)	.330 .484
C3–4	SEA DEA	3 (5.1%) 11 (18.6%)	10 (7.4%) 45 (33.3%)	.552 <.05	9 (9.8%) 19 (20.7%)	8 (12.7%) 25 (39.7%)	.568 <.05
C4–5	SEA DEA	6 (10.2%) 21 (35.6%)	31 (23.0%) 64 (47.4%)	<.05 .127	11 (12.0%) 18 (19.6%)	21 (33.3%) 29 (46.0%)	<.05 <.05
C5–6	SEA DEA	7 (11.9%) 27 (45.8%)	35 (25.9%) 84 (62.2%)	<.05 <.05	11 (12.0%) 31 (33.7%)	15 (24.7%) 39 (61.9%)	<.05 <.05
C6–7	SEA DEA	15 (25.4%) 23 (39.0%)	51 (37.8%) 79 (58.5%)	.095 <.05	19 (20.7%) 33 (35.9%)	23 (36.5%) 40 (63.5%)	<.05 <.05

Values in parentheses represent percentage of patients with SFI either as < cutoff or ≥ cutoff.

DEA = deep extensor, SEA = superficial extensor, SFI = subcutaneous fat index.

**Table 4:** Modic change and Intervertebral disk degeneration status of the cervical spine according to SFI cutoff value.

	<65 men		≥65 women	
	<8.49 mm	≥8.49 mm	<12.63 mm	≥12.63 mm
For Modic change				
C2–3	1 (1.0%)	1 (0.5%)	2 (8.6%)	2 (4.4%)
C3–4	4 (4.3%)	22 (11.7%)	2 (8.6%)	4 (8.8%)
C4–5	8 (8.6%)	25 (13.2%)	5 (21.7%)	9 (20.0%)
C5–6	13 (13.9%)	44 (23.4%)	9 (39.1%)	20 (44.4%)
C6–7	5 (5.3%)	24 (12.7%)	5 (21.7%)	10 (22.2%)
For IVDD C2–3 C3–4 C4–5 C5–6 C6–7	0 13 (13.9%) 15 (16.1%) 13 (13.9%) 11 (11.8%)	0 24 (12.7%) 49 (26.0%) 57 (30.3%) 41 (21.8%)	0 1 (4.3%) 3 (8.7%) 4 (10.9%) 0 (3.3%)	0 6 (13.3%) 9 (20.0%) 13 (28.8%) 5 (11.1%)

Values in parentheses represent percentage of patients with SFI either as < cutoff or ≥ cutoff.

IVDD = intervertebral disk degeneration, SFI = subcutaneous fat index.

## 4. Discussion

The goal of the present study was to explore the relationship between cervical spine SFTT, NP, and spinal deterioration. The SFTT values at the C3 level yielded the highest AUC value when used to differentiate between NP patients and asymptomatic controls. These are the first published analyses to our knowledge specifically examining the link between NP, cervical degeneration, and specific cervical SFTT threshold values. These analyses ultimately revealed that the SFI was able to reliably differentiate between NP patients and asymptomatic controls irrespective of gender.

Obesity has long been suggested to represent an important risk factor associated with the incidence of NP, Modic changes, IVDD, and fatty infiltration of the paraspinal muscles,^[12,21,22]^ BMI has been used as an indicator of obesity for over a century, and at baseline higher BMI values are related to an increase in NDI (Neck Disability Index) but unrelated to JOA (Japanese Orthopaedic Association Scores), with similar results at 1 year post-surgery.^[23]^ However, the utility of BMI values is limited as they fail to consider patient age, sex, muscle mass, bone structure, or fat distribution characteristics.^[24]^ BMI does not provide a reliable means of evaluating local adiposity in the neck region, and its value is further hampered by the distinct patterns of fat deposition observed in men and women.^13^ In their multivariate analysis of 3671 and 400 patients that respectively underwent anterior cervical fusion and posterior cervical fusion, Rafael et al detected no significant relationship between obesity and complications within 30 days postoperatively.^[25]^ The present study was developed to explore the relationship between SFTT values at multiple cervical spinal levels, ultimately revealing that the SFTT at the C3 level was the best analyzed predictor of NP. These results are inconsistent with those reported previously by Yavuz et al,^[11]^ who found the CFTT at the C5 level to be related to cervical degeneration. While the SFTT at the C5 level was able to significantly differentiate between NP patients and controls in this study, the AUC value for SFTT at the C3 level was higher. This may be because the C3 vertebrae are less susceptible to positional changes. These results indicated that males with an SFTT >8.21 mm and women with an SFTT >9.64 mm were more likely to experience symptoms of NP. Those patients exceeding these SFTT cutoff thresholds were also more likely to exhibit IVDD, Modic changes, and fatty infiltration of the paravertebral muscle.

A growing body of evidence suggests that increased body weight and higher BMI are risk factors related to IVDD incidence.^[26,27]^ Obesity can influence the spine through both inflammation and increased mechanical loading.^[12]^ Consistently, IVDD is a highly prevalent issue among patients with metabolic syndromes including obesity and type 2 diabetes, both of which entail the role of adipokines as key regulators of fibrosis, the degradation of the extracellular matrix, and inflammatory activity.^[28]^ A range of metabolic abnormalities are evident in obese individuals including dysregulated lipid and insulin homeostasis, chronic inflammation, excessive fat storage, and dysregulation of the white adipose tissue.^[29]^ Overweight and obese individuals generally exhibit more severe IVDD than do individuals with normal weight and adiposity.^[30]^ Cross-sectional research indicates that higher BMI values are closely related to disc space narrowing and the degree of lumbar IVDD,^[31]^ although gender-related variations in these relationships are not consistent across reports.^[32,33]^ The impact of fat tissue on cervical spine deterioration, however, is a topic that warrants further study.

It has long been thought that overweight and obesity contribute to pain due to increased physical stress on the body. However, recent evidence indicates that it could be related to metabolic factors and systemic chronic inflammation.^[34]^ Studies show that fat mass is linked to markers of inflammation in people who are overweight or obese. This suggests that subcutaneous fat thickness could be an important factor in NP. Our research has demonstrated the critical part that fat mass plays in pain and has identified the amount of subcutaneous fat thickness that is necessary to cause NP. It is possible that physical loading and metabolic effects may be related to obesity-induced pain. Excess loading can lead to changes in body mechanics, posture, and gait, creating an unfavorable biomechanical environment.^[35]^ Previous studies have shown that obese individuals have higher levels of cytokines and inflammatory markers, such as C-reactive protein, Interleukins-6, tumor necrosis factor-alpha.^[36]^ This could mean that people with high levels of fat mass have a higher chance of having peripheral or central sensitization due to increased systemic inflammation, which could lead to more painful sites.

There are some limitations to this study. As this was a retrospective analysis, causality with respect to the association between obesity and spinal deterioration could not be established. In addition, as the recruited patients were from outpatient and physical examination centers, certain demographic details with the potential to influence cervical spine deterioration such as smoking history, history of alcohol use, education level, BMI, and lifestyle factors were not available for analysis. In order to address these limitations, future studies should consider conducting longer-term follow-up trials involving multi-center cohorts from different ethnic populations. Additionally, only SFTT values of the cervical spine as determined based on MRI images were analyzed herein, and some prior reports have suggested that cervical deterioration is also related to both neck circumference and the degree of paravertebral muscle fat infiltration. Accordingly, future studies should focus on the potential relationship between these variables and NP.

## 5. Conclusion

The present results indicate that the SFI was able to reliably differentiate between NP patients and asymptomatic control individuals. Both male and female patients with an SFTT at the C3 level that was above the defined gender-specific cutoff level exhibited higher degrees of Modic changes, IVDD, and fatty infiltration of the paravertebral muscles. These results are noteworthy as they suggest that elevated adipose tissue thickness in the neck region may be linked to the risk of cervical degeneration.

## Author contributions

**Conceptualization:** Jian Cao, Dong Sun, Peng Liu.

**Data curation:** Dong Sun, Lianzhi Guo.

**Formal analysis:** Jian Cao.

**Investigation:** Lianzhi Guo.

**Methodology:** Lianzhi Guo.

**Supervision:** Rui Wang.

**Validation:** Rui Wang.

**Writing – original draft:** Jian Cao, Peng Liu.

**Writing – review & editing:** Jian Cao, Peng Liu.
